# COVID-19-Induced Cardiomyopathy in a Young Nulliparous Woman

**DOI:** 10.7759/cureus.16516

**Published:** 2021-07-20

**Authors:** Katherine Chenevey, William Murdoch, A. Vincent Songco

**Affiliations:** 1 Family Medicine Residency, ProMedica Monroe Regional Hospital, Monroe, USA; 2 Cardiology, ProMedica Monroe Regional Hospital, Monroe, USA

**Keywords:** coronavirus-19, covid, cardiomyopathy, nonischemic, cardiac imaging

## Abstract

A young nulliparous woman presented with new-onset heart failure several weeks after an asymptomatic coronavirus disease 2019 (COVID-19) infection. Investigation revealed non-ischemic cardiomyopathy without apical ballooning. A literature review demonstrates other reported cases of COVID-19-induced cardiomyopathy; however, our patient was demographically unique from previously described cases, presented later, and did not have Takotsubo-like findings. Our patient continued to have evidence of cardiac remodeling and a reduced ejection fraction months after presentation. This case highlights the importance of surveilling for the post-infectious sequelae of COVID-19, along with the wide demographic range of patients susceptible to such outcomes.

## Introduction

The coronavirus disease 2019 (COVID-19) pandemic has infected 116 million individuals worldwide and caused 2.58 million deaths, as of March 2021 [1]. Although most patients fully recover, and few experience long-term sequelae, it is important for physicians to be aware of and recognize potential short- or long-term cardiac effects of infection with the COVID-19 virus. We performed a literature review to establish what was already known about myocarditis in the setting of COVID-19. We conducted a PubMed search with the keywords “COVID,” “cardiomyopathy,” either “case study” or “case series” and either “MRI,” “systolic dysfunction,” “reduced ejection fraction,” or “depressed ejection fraction.” This search yielded 17 unique articles describing a total of 26 patients. The majority of the reports addressed patients with active COVID-19 respiratory symptoms with concurrent findings supporting the diagnosis of myocarditis. This article was previously presented as a meeting abstract at the 1st Southeast Michigan Research Day on June 2, 2021.

Early COVID-19 cardiomyopathy case reports centered on Takotsubo cardiomyopathy as sequelae of the disease, sometimes associated with stroke [2-3]. One case report identifies two patients with Takotsubo cardiomyopathy in the setting of acute COVID infection who subsequently underwent cardiac catheterization; both patients were noted to have normal coronary arteries. The first patient had basal segment akinesia on angiography. The second patient had presented with chest pain and lateral ST-segment elevation on ECG; she was found to have a moderate reduction in systolic function [4]. Another case found a Takotsubo post-COVID effect in a middle-aged woman [5]. Patients with Takotsubo cardiomyopathy after COVID sometimes had significant improvement within a short time frame [6].

Two of the most relevant articles describe late acute severe myocarditis in middle-aged male patients without cardiac history, both of whom presented with dyspnea six weeks after developing acute COVID-10 symptoms. Both patients were found to have elevation in troponin T, and cardiac MRI demonstrated reduced LVEF [7-8].

Another common demographic theme in these case reports was the presence of peripartum patients. One describes a woman with flu-like symptoms one week prior to delivery; she developed dyspnea four weeks after delivery and was found to be COVID-19 positive on polymerase chain reaction (PCR) with elevated troponin and depressed systolic function confirmed on cardiac MRI [9]. An additional case series describes two pregnant patients with normal troponin levels but newly identified reduced ejection fraction; one developed symptoms consistent with myocarditis during labor at 39 weeks, the other developed symptoms at 33 weeks, and labor was induced secondary to clinical decline [10].

Radiographic surveillance of post-COVID patients has demonstrated that up to 30% may have some evidence of left ventricular dysfunction on cardiac MRI at three months [11] and that many of these patients are asymptomatic from a cardiac standpoint [12].

## Case presentation

Ms. N is a 23-year-old nulliparous woman with morbid obesity (body mass index (BMI) 40.3 kg/m^2^) who presented to the emergency room at our local community hospital. Her concerns were progressive exertional shortness of breath for two weeks and tachycardia for three days. She had access to a home pulse oximeter and had noted heart rates in the 120s with normal oxygen levels. Ms. N did admit to some mild shortness of breath at baseline but stated that the recent progression was much worse. The patient noted recent past medical history significant for COVID-19 infection that occurred six weeks prior to presentation, diagnosed by nasal swab. Of note, she was an employee at a local nursing home and believes she contracted the virus at the facility. Ms. N stated that the acute illness was treated at home with symptomatic treatment and that her COVID-19 symptoms of cough and fever had completely resolved following the acute illness. Her past medical history was otherwise significant for asthma, depression, and migraines. Her only regular medication was an oral contraceptive. She did relate a family history of heart disease but was not able to specify further.

**Figure 1:**
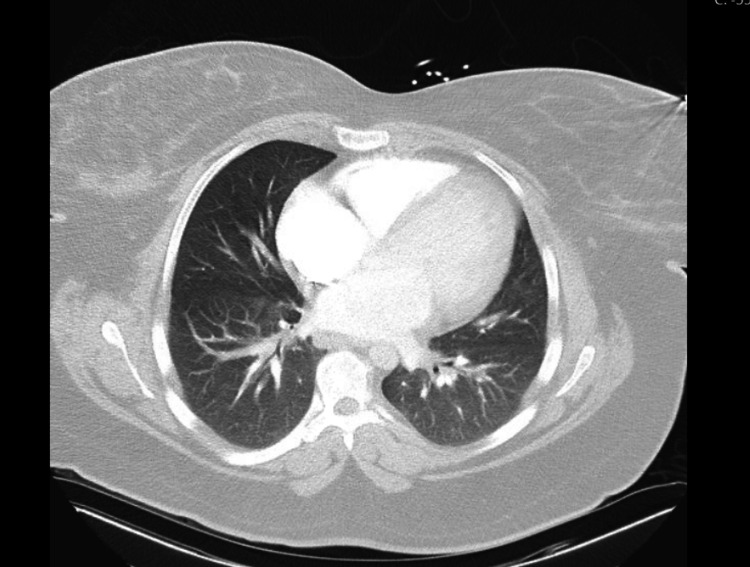
CT angiogram obtained on admission

At the time of the emergency room presentation, Ms. N was noted to have sinus tachycardia and tachypnea. Her brain natriuretic peptide (BNP) was elevated at 1082 pg/mL while serum myoglobin and troponin-I were normal. The patient’s COVID-19 immunoglobulin G (IgG) was positive. Pertinent presenting labs are presented in Table 1. Computed tomography (CT) of her chest revealed the absence of pulmonary embolism but the presence of multifocal lymphadenopathy throughout the chest. A representative image is shown in Figure 1.

**Table 1:** Laboratory data Laboratory data from Ms. N's presentation at ProMedica Monroe Regional Hospital TSH: thyroid-stimulating hormone; BNP: brain natriuretic peptide; SARS-CoV-2: severe acute respiratory syndrome coronavirus 2; THC: tetrahydrocannabinol

Laboratory Value	Normal Values	Hospital Presentation
White Blood Cells (10^9^/L)	4.0-11.0	11.8
Creatinine (mmol/L)	0.40-1.00	1.03
Troponin I (ng/mL)	0.00-0.04	0.01
TSH (uIU/mL)	0.49-4.67	1.98
BNP (pg/mL)	<100	1082
SARS-CoV-2 Rapid	Negative	Negative
SARS-CoV-2 IgG	Negative	Positive
Urine Drug Screen, 10 Metabolite	Negative	Positive for THC
Urine Pregnancy	Negative	Negative
Myoglobin (ng/mL)	14.3-65.8	34.8

EKGs revealed a normal sinus rhythm and were unchanged during her hospital stay. She was admitted to the hospital with a provisional diagnosis of acute decompensated systolic congestive heart failure, and the cardiology service was consulted. Presumptive heart failure treatment was initiated with valsartan, metoprolol succinate, and furosemide. Echocardiography revealed severely decreased systolic function with an ejection fraction of 10%-15%, grade I diastolic dysfunction, and severe global hypokinesis. Cardiac catheterization demonstrated normal coronary arteries and elevated left ventricular end-diastolic pressure of 38 mmHg. The consultant cardiologist believed Ms. N’s cardiomyopathy to have been due to her recent COVID-19 diagnosis.

Ms. N was managed as an inpatient with diuresis with an improvement in her presenting symptoms. She was discharged to home with prescriptions for furosemide, metoprolol succinate, and valsartan. The interval echocardiogram at one month redemonstrated an ejection fraction of 10-15%, and the patient was referred to cardiopulmonary rehab. Ms. N was also referred to a specialized heart failure clinic in our healthcare system; the cardiologist there believed that she had a pre-existent cardiac remodeling process that was made worse by her COVID-19 infection. The heart failure clinic ordered a repeat CT of the patient’s chest, which redemonstrated the bilateral hilar and mediastinal lymphadenopathy. Cardiac MRI performed two months after hospital discharge demonstrated a dilated left ventricle without ventricular wall hypertrophy; this was interpreted by the consultant radiologist and cardiologist as consistent with nonischemic cardiomyopathy.

## Discussion

Ms. N’s case of COVID-19-associated myocarditis represents a unique addition to what is already known about the potential sequelae of a COVID-19 infection. Ms. N was notably different in her demographics from previously published cases. She was young, previously took no daily medications, and had an uncomplicated past medical history without prior cardiac issues. Relative to other cases published thus far, she was in overall better health and would have been expected to have fewer COVID-19-related comorbidities. As previously mentioned, she was nulliparous, removing the confounding factor of possible peripartum cardiomyopathy as a cause of her acute heart failure.

Also somewhat unique to Ms. N’s case was the six-week latency between her first positive COVID-19 test and her presentation to the hospital for heart failure; the majority of patients currently reported in the literature were found to have heart failure concurrent with initial admissions for other COVID-19 symptoms with positive COVID-19 PCR testing. Ms. N’s positive COVID-19 IgG and negative COVID-19 PCR strengthen the differential diagnosis that this was myocarditis that developed subsequent to the initial viral infection, rather than heart failure occurring during the acute respiratory phase of the disease. The pathophysiology of COVID-19-induced cardiomyopathy is not well understood, although virus effects on angiotensin-converting enzyme 2 (ACE2) receptor-expressing cells have been proposed as a possible mechanism [13].

Though it is not possible to say exactly when Ms. N’s heart failure started relative to the date of her presentation to the emergency department, it was notable that her cardiac enzymes were negative at the time of and throughout her hospital admission. With the exception of one of the pregnant patients described in the article by Juusela et al. (2020), all patients identified during the literature review had clinically significant elevations in troponin and/or myoglobin.

While the initial differential diagnosis for a young patient with new-onset heart failure is broad, Ms. N’s hospital evaluation did help to narrow this differential considerably. Initial testing indicated negative pregnancy status, and urine drug testing excluded multiple street drugs as possible causes of heart failure. Ms. N’s diagnostic evaluation was further strengthened by her negative cardiac catheterization, which was omitted in the majority of similar case reports that were reviewed.

## Conclusions

Ms. N’s case further expands on what is known about myocarditis as a possible sequela of COVID-19 infection. As COVID-19 case numbers continue to increase globally, it may reasonably be expected that additional patients may present in a similar fashion, and clinicians must be aware of this possible complication as part of their differential diagnosis of patients with a recent COVID-19 illness. As more is known about this complication, clinicians and researchers might better identify and differently manage patients in the acute phase who are at greater risk of developing myocarditis. As additional reports of COVID-19 “long haulers” surface, additional data is also needed on how best to manage patients with COVID-19-associated myocarditis in the long term.
